# Bone density and depressive disorder: a meta‐analysis

**DOI:** 10.1002/brb3.489

**Published:** 2016-05-18

**Authors:** Julietta Ursula Schweiger, Ulrich Schweiger, Michael Hüppe, Kai G. Kahl, Wiebke Greggersen, Eva Fassbinder

**Affiliations:** ^1^ Department of Psychiatry and PsychotherapyLübeck University Medical SchoolLübeckGermany; ^2^ Department of AnesthesiologyLübeck University Medical SchoolLübeckGermany; ^3^ Department of Psychiatry, Social Psychiatry and PsychotherapyHannover Medical SchoolHannoverGermany

**Keywords:** Absorptiometry, bone density, depressive disorder, meta‐analysis, osteoporosis, photon

## Abstract

**Background:**

The aim of this study was to evaluate the evidence of low bone mineral density (BMD) in depression. Low BMD is a major risk factor for osteoporotic fractures and frailty.

**Methods:**

The searched database was Pubmed, Meta‐analysis included human studies in men and women fulfilling the following criteria: (1) assessment of BMD in the lumbar spine, the femur or the total hip; (2) comparison of BMD between depressed individuals and the healthy control group; (3) measurement of BMD using dual‐energy X‐ray absorptiometry (DEXA); and (4) data on the mean, standard deviation, or standard error of BMD.

**Results:**

Twenty‐one studies were identified, encompassing 1842 depressed and 17,401 nondepressed individuals. Significant negative composite weighted mean effect sizes were identified for the lumbar spine (*d* = −0.15, 95%CL −0.22 to −0.08), femur (*d* = −0.34, 95%CL −0.64 to −0.05), and total hip (*d* = −0.14, 95%CL −0.23 to −0.05) indicating low BMD in depression. Examining men and women shows low bone density in the lumbar spine and femur in women and low bone density in the hip in men. The differences between men and women with MDD and the comparison group tended to be higher when examined by expert interviewers. Low bone density was found in all age groups.

**Conclusions:**

Bone mineral density is reduced in patients with depressive disorders. The studies provide little evidence for potential relevant mediating factors.

## Background

Depression is a common chronic debilitating disease associated with mood and cognitive and physical symptoms (Pratt and Brody 2014). Major depressive disorder (MDD) is one of the leading causes of years lived with disability in most countries (Global Burden of Disease Study, Collaborators 2015). The risk of developing MDD is twice as high for women as for men (Kessler and Bromet 2013). Osteoporosis is a highly prevalent degenerative bone disease (Wade et al. 2014) and is characterized by low bone mineral density (BMD) and the deterioration of the skeletal structure (Golob and Laya 2015). It is associated with an increased risk of fracture. In 1994, the first observation that MDD is associated with low lumbar bone mineral density was published (Schweiger et al. 1994). In the following years, many studies that reexamined this effect were published. The results are heterogeneous depending on the study design, the evaluation of depression, and other characteristics of the included population.

There are many possible reasons why depression may be associated with low BMD. Patients with depression show dysregulation of the HPA system function. There is altered secretion of corticotropin‐releasing hormone, corticotropin and cortisol, and altered regulation of glucocorticoid and mineralocorticoid receptor signaling (Huang and Lin 2015). Cortisol is known to influence bone loss (Marques et al. 2009). Proinflammatory cytokines have been found to be associated with depressive disorders (Valkanova et al. 2013; Young et al. 2014). Studies have shown that elevated serum concentrations of IL‐1, IL‐6, TNF‐*α*, CRP, and MCP‐1 are present in depressed patients (Young et al. 2014). Recent studies have shown that cytokines and immune cells may have a direct osteoclastogenic effect (Jung et al. 2014) and also activate the HPA axis, with the resultant release of glucocorticoids. MDD may result in behavior changes, such as increased alcohol and nicotine intake, unhealthy nutrition, and decreased physical activity (Brot et al. 1999; Dorn et al. 2011; Cohn et al. 2014; Alghadir et al. 2015; Bailey and van Wijngaarden 2015; Kim et al. 2015; Villareal et al. 2016). The treatment of depression with antidepressants, such as tricyclic antidepressants (TCAs) or selective serotonin reuptake inhibitors (SSRIs), may also exert negative effects on bone health (Diem et al. 2013a; Sheu et al. 2015).

In particular, SSRIs were associated with an increased risk of fractures (Sheu et al. 2015). Whether SSRIs are associated with bone loss is controversial (Diem et al. 2013a). The specific biochemical nature of serotonergic pathways that influence bone metabolism remains still unclear. Serotonin receptors (5‐HT) and 5‐HT transporters (5‐HTT) were identified in many major bone cell types. A polymorphism in the promoter region of the serotonin transporter gene (5‐HTTLPR) is assumed to moderate the relationship between stress and depression (Karg et al. 2011). However, there are no data available on how this polymorphism affects BMD in the general population. SSRIs selectively and potently block 5‐HTT. This results in a higher concentration of free circulating 5‐HT, which may have negative effects on bone metabolism. In vitro studies showed that mice with a null mutation in the 5‐HTT gene or mice treated with SSRIs exhibit reduced BMD, altered skeletal architecture, and reduced bone mechanical properties (Bliziotes 2010; Warden et al. 2010).

An increased risk of osteoporosis is of high importance in patients with MDD because it may lead to an increased fracture rate and premature frailty. In its early stages, it is a symptom‐free disease and is undetectable in routine examinations. The knowledge of the association between depression and low BMD is important for several reasons. It affects counseling of patients with MDD with respect to exercise, nutrition, smoking, and alcohol consumption. It affects the determination of the necessity of diagnostic osteodensitometry and the need of treatment for osteoporosis.

This meta‐analysis examined the state of research on the relationship between MDD and BMD in humans. Earlier comprehensive reviews date from before 2010 (Mezuk et al. 2008; Yirmiya and Bab 2009). The aim was to perform a meta‐analysis on all relevant studies comparing patients with depression to an appropriate comparison population with respect to BMD.

## Methods

### Sample of studies

**Figure 1:**
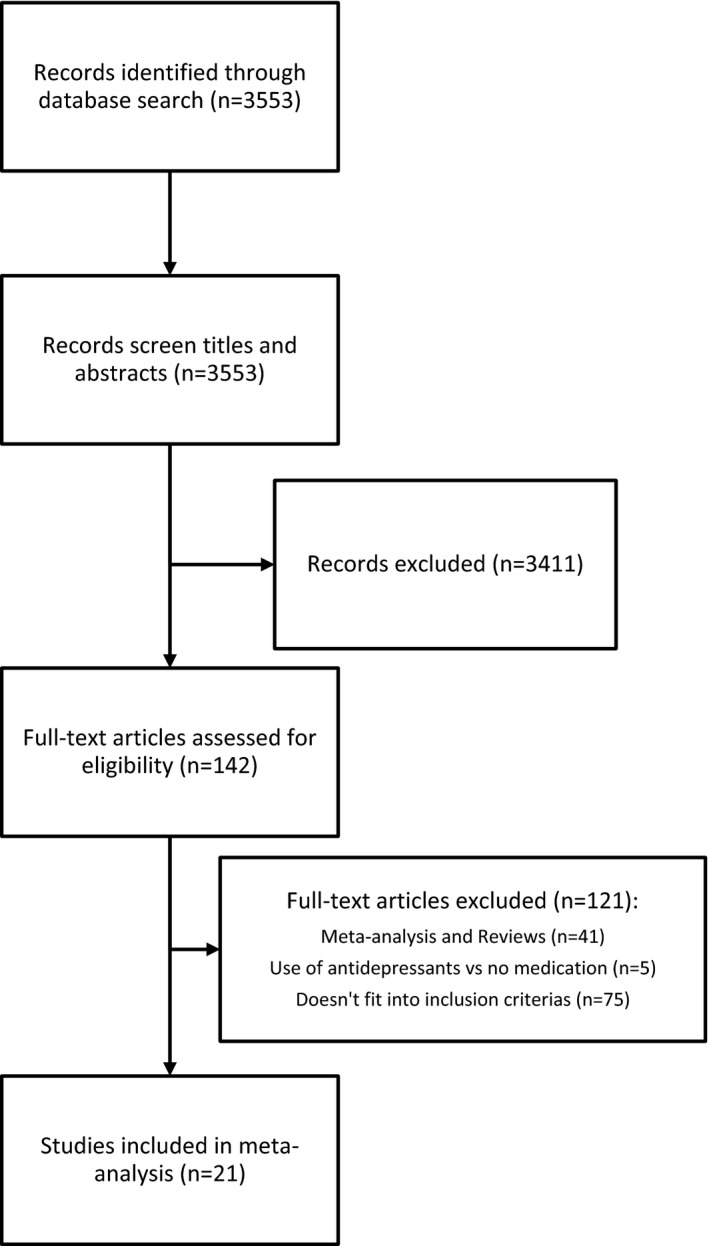
Diagram depicting the flow of information in the meta‐analysis according to PRISMA statement.

The meta‐analysis followed the frame provided by the PRISMA statement (Moher et al. 2009). Figure 1 provides the flowchart. Studies were identified using the computerized database MEDLINE, covering the period from its inception to May 15, 2015. A comprehensive literature search of MEDLINE was conducted without language restrictions and with the search terms *bone* and *depression;* results were restricted to human studies. We reviewed each title and abstract of articles to exclude obviously irrelevant publications. Relevant reports were also double checked with the references list of published articles, including several reviews, with no additional identified records. Inclusion criteria were as follows: (1) assessment of BMD in the lumbar spine, the femur, or the total hip, (2) comparison of BMD between depressed individuals and a healthy control group, (3) measurement of BMD using dual‐energy X‐ray absorptiometry (DEXA), and (4) data on the mean, standard deviation, or standard error of BMD. In total, 21 articles complied with these criteria (Michelson et al. 1996; Amsterdam and Hooper 1998; Whooley et al. 1999, 2004; Robbins et al. 2001; Kavuncu et al. 2002; Yazici et al. 2003, 2005; Jacka et al. 2005; Konstantynowicz et al. 2005; Ozsoy et al. 2005; Wong et al. 2005; Altindag et al. 2007; Diem et al. 2007, 2013b; Eskandari et al. 2007; Petronijevic et al. 2008; Charles et al. 2012; Cizza et al. 2012; Fazeli et al. 2013; Sommerhage et al. 2013).

### Data extraction

Data were extracted by two examiners (JUS and US) using standardized data abstraction forms. The extracted information included (1) the author's names, (2) year of publication, (3) country where the study was conducted, (4) sample size of the patient and the control groups, (5) gender, (6) age, (7) menopausal status, (8) medication use, (9) depression assessment tool, and (10) BMD, T score, and Z score of the lumbar spine, femur, and total hip. In two reports (Altindag et al. 2007; Cizza et al. 2012), the standard deviation was particularly small and the *P*‐value did not fit. We conservatively assumed that there was confusion of standard deviation and standard error, and we converted this value into standard deviation.

### Statistical analyses

We carried out several meta‐analyses for BMD in depressed and nondepressed individuals. Analyses were performed with Comprehensive Meta‐Analysis (CMA) software (Englewood, New Jersey). In each meta‐analysis, standardized effect sizes derived from the individual studies were combined to determine a composite mean weighted effect size along with its 95% confidence interval (CI) and significance level (i.e., the effect size is significant if the CI does not include a zero). Greater weight is given to studies with larger samples; hence, this procedure corrects for bias with small sample sizes. Because the effects of depression on BMD were studied in different settings (e.g., depression diagnosed by an expert or self‐reported) and because participants’ demographic data differed greatly between studies, we assumed the presence of heterogeneity a priori – that the effect of individual trials would vary more than expected by chance alone. Therefore, the variance and statistical significance of differences were assessed with random‐effect calculations in all analyses. To determine the validity of the meta‐analysis, we employed funnel plots (i.e., plots of the standard difference in means [*d*] against the SEM). This was followed by quantitative evaluation of the degree of asymmetry. (Borenstein et al. 2009). The analyses were independently made for the following bones: lumbar spine, femur, and total hip. For each bone, all associated studies were pooled and individually analyzed for females and males.

## Results

A total of 3553 records were identified through this search. Approximately 142 full‐text articles were assessed for eligibility. Of the 21 studies, five included females and males, 13 had only females, and three had only male participants. The studies encompassed 1842 depressed and 17,401 nondepressed individuals (Table 1).

**Table 1:** Characteristics of all studies that compared bone mineral density in men and women with and without depression using dual‐energy X‐ray absorptiometry (DEXA)

Study name	Year	Country	Depression/Control Subjects (*n*)	Gender	Age (Depression/Control)	Menopause status	Depression assessment tool	Bone site
S.J. Diem et al.	2013b	US	136/2328	m	76.7/75.7		Self‐rating, GDS	Hip
P.K. Fazeli et al.	2013	US	17/16	f	16.6/16.3	Pre	Expert opinion + CDRS‐R	Spine, femur, hip
P.K. Fazeli et al.	2013	US	16/16	m	16.8/16.4		Expert opinion + CDRS‐R	Spine, femur, hip
V. Sommerhage et al.	2013	Estonia	50/30	f	50/46	Pre & post	Expert Interview MINI	Spine, femur
L.E. Charles et al.	2012	US	14/13	f	Unclear	Pre & post	Self‐rating, CES‐D	Spine, femur, hip
L.E. Charles et al.	2012	US	17/20	m	Unclear		Self‐rating, CES‐D	Spine, femur, hip
G. Cizza et al.	2012	US	92/44	f	36.0/35.3	Pre	Expert Interview SCID	Spine, Femur, Hip
M. Petronijevic et al.	2008	Serbia	73/47	f	40.7/40.5	Pre	Expert interview, HAMD	Spine, femur
O. Altindag et al.	2007	Turkey	36/41	f	39.8/42.8	Pre	Expert interview, HAMD	Spine, femur
S. Diem et al.	2007	US	200/3977	f	76.7/75.6	Post	Self‐rating, GDS	Hip
F. Eskandari et al.	2007	US	89/44	f	35/35	Pre	Expert interview, HAMD	Spine, femur, hip
F.N. Jacka et al.	2005	Australia	14/64	f	50.5/53.4	Peri	Self‐rating	Spine, hip
J. Konstantynowicz et al.	2005	Poland	14/31	f	17/16.5	Pre	Expert interview, HAMD	Spine
S. Özsoy et al.	2005	Turkey	21/23	f	37.57/33.37	Pre & post	Expert interview, MADRS	Spine, femur
S. Özsoy et al.	2005	Turkey	21/11	m	37.6/33.7		Expert interview, MADRS	Spine, femur
S.Y.S. Wong et al.	2005	Hong Kong	169/1830	m	72.94/72.34		Expert opinion	Spine, hip
A.E. Yazici et al.	2005	Turkey	35/30	f	44.8/46.2	Pre	Expert interview, HAMD	Spine, femur
M. A. Whooley et al.	2004	US	16/499	m	64.6/66.7		Self‐rating, GDS	Spine, hip
K.M. Yazici et al.	2003	Turkey	25/15	f	30.8/31.2	Pre	Expert interview, HAMD	Spine, femur
V. Kavuncu et al.	2002	Turkey	42/42	f	35.4/36.7	Pre	Expert interview, HAMD	Spine, femur
J. Robbins et al.	2001	US	248/1304	m/f	74.9/74.2		Self‐rating, CES‐D	Hip
M.A. Whooley et al.	1999	US	467/6947	f	74.5/73.3	Post	Self‐rating, GDS	Spine, hip
J.D. Amsterdam et al.	1998	US	4/3	f	38/37	Pre & post	Expert interview, HAMD	Spine
J.D. Amsterdam et al.	1998	US	2/2	m	48/39		Expert interview, HAMD	Spine
D. Michelson et al.	1996	US	24/24	f	41/41	Pre & post	Expert interview, SCID	Spine, femur

### Lumbar spine

**Figure 2:**
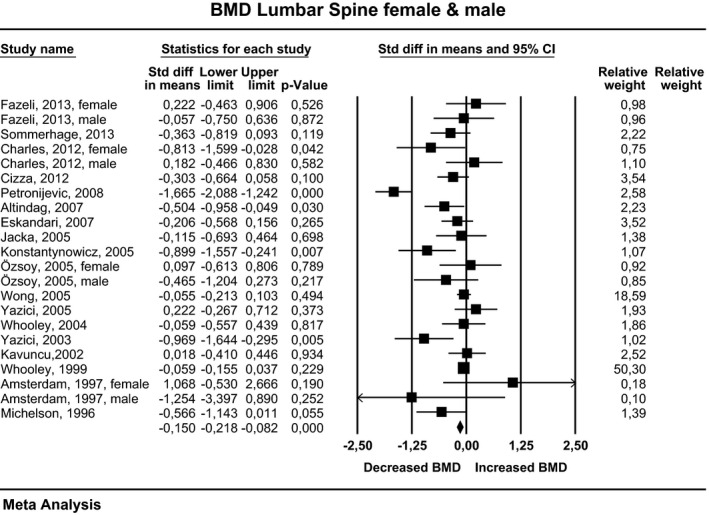
Estimates of all studies that compared bone density in the lumbar spine in men and women with and without depression. The diamond at the bottom of the graph denotes the overall estimate of the association between depression and lumbar spine bone density.

Eighteen studies examined the lumbar spine; in four studies, data on females and males were shown separately. The effect sizes pooled for females and males, corresponding CI, *P*‐values, and relative weights for each study and a forest plot summarizing the association between depression and BMD are shown in Figure 2. The effect sizes ranged from −1.67 to 1.07, with 16 studies reporting either decreased or unchanged BMD and six studies showing increased BMD. The composite weighted mean effect size, *d*, was −0.30, and its CI was between −0.48 and −0.11; this implies that overall BMD is significantly lower in depressed than in nondepressed individuals (*P* = 0.001). Publication bias was assessed using the funnel plot procedure. There were nonsignificant rank correlation and regression intercept values. The fail‐safe number indicated that an additional 141 negative studies would be necessary for the present results to lose their significance.

For females, the effect sizes ranged from −1.67 to 1.07, with 11 studies reporting either decreased or unchanged BMD and five studies showing increased BMD. The composite weighted mean effect size, *d*, was −0.36, and its CI was between −0.60 and −0.11. This implies that overall BMD was significantly lower in depressed than in nondepressed women (*P* = 0.005). For males, the effect sizes ranged from −0.47 to 0.18, with five studies reporting either decreased or unchanged BMD and one study showing increased BMD. However, the composite weighted mean effect size, *d*, was −0.06, and its CI was between −0.21 and 0.08. This implies that overall BMD was not significantly different in depressed and nondepressed men (*P* = 0.369).

In 10 studies, only premenopausal women were examined. The effect size ranged from −1.67 to 0.22, with seven studies reporting either decreased or unchanged BMD and three studies showing increased BMD. The composite weighted mean effect size, *d*, was −0.41, and its CI was between −0.80 and −0.042. This implies that overall BMD was significantly different in depressed and nondepressed premenopausal women (*P* = 0.030).

In 14 studies, depression was evaluated by expert interviews; in three studies, data on females and males were shown separately. When pooled for females and males, the effect sizes ranged from −1.67 to 1.07, with 12 studies reporting either decreased or unchanged BMD and five studies showing increased BMD. The composite weighted mean effect size, *d*, was −0.35, and its CI was between −0.60 and −0.09. This implies that overall BMD is significantly lower in depressed individuals evaluated by an expert than in nondepressed participants (*P* = 0.007). For females, the effect sizes ranged from −1.67 to 1.07, with eight studies reporting either decreased or unchanged BMD and five studies showing increased BMD. The composite weighted mean effect size, *d*, was −0.37, and its CI was between −0.69 and −0.048. This implies that overall BMD was significantly lower in depressed women evaluated by an expert than in nondepressed women (*P* = 0.024). For males, the effect size ranged from −1.25 to −0.05, with all four studies reporting either decreased or unchanged BMD. However, the composite weighted mean effect size, *d*, was −0.08, and its CI was between −0.22 and 0.07. This implies that the overall BMD was not significantly different in depressed men evaluated by an expert than in nondepressed men (*P* = 0.309).

In three studies, depression was self‐reported; in two studies, data for females and males were shown separately. When pooled for females and males, the effect sizes ranged from −0.81 to 0.18, with four studies reporting either decreased or unchanged BMD, and one study showing increased BMD. However, the composite weighted mean effect size, *d*, was −0.07, and its CI was between −0.18 and 0.04, implying that overall BMD was not significantly different in depressed patients evaluated by self‐reports and nondepressed participants (*P* = 0.217). For females, the effect sizes ranged from −0.81 to −0.06, with all three studies reporting either decreased or unchanged BMD. However, the composite weighted mean effect size, *d*, was −0.18, and its CI was between −0.52 and 0.16; this implies that the overall BMD was not significantly different in depressed women evaluated by a self‐reported test and nondepressed women (*P* = 0.295). For males, the effect sizes ranged from −0.06 to 0.18, with one study reporting either decreased or unchanged BMD and one study reporting an increase in BMD. However, the composite weighted mean effect size, *d*, was −0.03, and its CI was between −0.36 and 0.43. This implies that the overall BMD was not significantly different in depressed men evaluated by an expert and nondepressed men (*P* = 0.880).

### Femur

**Figure 3:**
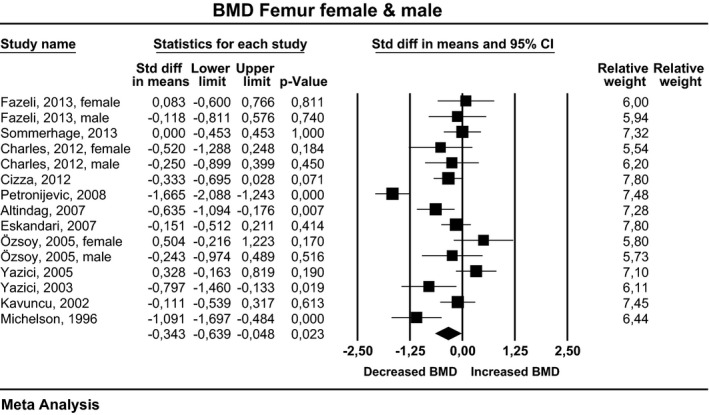
Estimates of all studies that compared bone density in the femur in men and women with and without depression. The diamond at the bottom of the graph denotes the overall estimate of the association between depression and femur bone density.

Twelve studies examined the femur. In three studies, data on females and males were shown separately. Effect sizes pooled for females and males, corresponding CI, *P*‐values, and relative weights for each study and a forest plot summarizing the association between depression and BMD are shown in Figure 3. The effect sizes ranged from −1.67 to 0.50, with 12 studies reporting either decreased or unchanged BMD and three studies showing increased BMD. The composite weighted mean effect size, d, was −0.34, and its CI was between −0.64 and −0.05. This implies that the overall BMD was significantly lower in depressed than in nondepressed participants (*P* = 0.023). Publication bias was assessed using the funnel plot procedure. The rank correlation and regression intercept values were nonsignificant. The fail‐safe number indicates that an additional 85 negative studies would be necessary for the present results to lose their significance.

For females, the effect size ranged from −1.67 to 0.50, with nine studies reporting either decreased or unchanged BMD and three studies showing increased BMD. The composite weighted mean effect size, *d*, was −0.37, and its CI was between −0.72 and −0.023. This implies that the overall BMD was significantly lower in depressed than in nondepressed women (*P* = 0.037). For males, the effect size ranged from −0.25 to 0.12, with all three studies reporting either decreased or unchanged BMD. However, the composite weighted mean effect size, *d*, was −0.20, and its CI was between −0.60 and 0.19. This implies that the overall BMD was not significantly different in depressed and nondepressed men (*P* = 0.314).

In eight studies, only premenopausal women were examined. The effect sizes ranged from −1.67 to 0.328, with six studies reporting either decreased or unchanged BMD and two studies showing increased BMD. However, the composite weighted mean effect size, *d*, was −0.42, and its CI was between −0.85 and 0.22. This implies that the overall BMD was not significantly different in depressed and nondepressed premenopausal women (*P* = 0.063).

In 11 studies, depression was evaluated by an expert interview; in two studies, females and males were both examined and were shown individually. When pooled for females and males, the effect sizes ranged from −1.67 to 0.33, with 10 studies reporting either decreased or unchanged BMD and three studies showing increased BMD. The composite weighted mean effect size, *d*, was −0.34, and its CI was between −0.67 and −0.01. This implies that the overall BMD was significantly lower in depressed than in nondepressed participants (*P* = 0.045). For females, the effect size ranged from −1.67 to 0.33, with seven studies reporting either decreased or unchanged BMD and four studies showing increased BMD. However, the composite weighted mean effect size, *d*, was −0.36, and its CI was between −0.73 and 0.01. This implies that the overall BMD was not significantly different in depressed women evaluated by an expert and nondepressed women (*P* = 0.057). For males, the effect sizes ranged from −0.24 to −0.12, with both studies reporting either decreased or unchanged BMD. However, the composite weighted mean effect size, *d*, was −0.18, and its CI was between −0.68 and 0.33. This implies that overall BMD was not significantly different in depressed men evaluated by an expert than in nondepressed men (*P* = 0.491).

In one study, depression was evaluated by self‐reports. In this study, data on females and males were shown separately. When pooled for females and males, the effect sizes ranged from −0.52 to −0.25. However, the composite weighted mean effect size, *d*, was −0.36, and its CI was between −0.86 and 0.13. This implies that the overall BMD was not significantly different in depressed individuals evaluated by self‐reports and nondepressed participants (*P* = 0.152).

### Total hip

**Figure 4:**
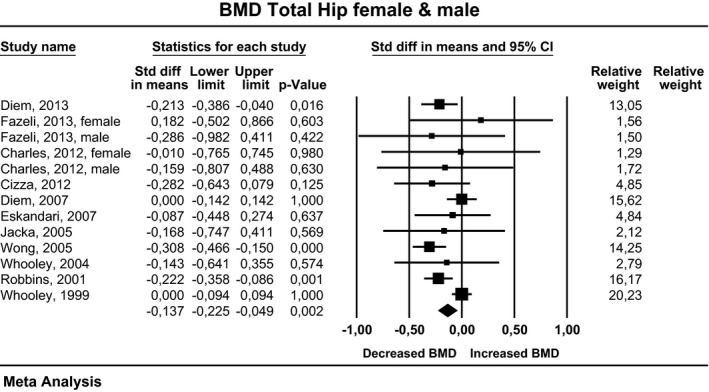
Estimates of all studies that compared bone density in the total hip in men and women with and without depression. The diamond at the bottom of the graph denotes the overall estimate of the association between depression and total hip bone density.

Eleven studies examined the total hip. In two studies, data on females and males were shown separately. When pooled for females and males, effect sizes, corresponding CI, *P*‐values, and relative weights for each study, and a forest plot summarizing the association between depression and BMD are shown in Figure 4. The effect sizes ranged from −0.31 to 0.18, with 12 studies reporting either decreased or unchanged BMD and one study showing increased BMD. The composite weighted mean effect size, d, was −0.14, and its CI was between −0.23 and −0.05. This implies that the overall BMD was significantly lower in depressed than in nondepressed participants (*P* = 0.002). Publication bias was assessed using the funnel plot procedure. The rank correlation and regression intercept values were nonsignificant. The fail‐safe number indicates that an additional 34 negative studies would be necessary for the present results to lose their significance.

For females, the effect sizes ranged from −0.28 to 0.18, with six studies reporting either decreased or unchanged BMD and one study showing increased BMD. However, the composite weighted mean effect size, *d*, was −0.02, and its CI was between −0.09 and 0.06. This implies that overall BMD was not significantly different in depressed and nondepressed women (*P* = 0.669). For males, the effect size ranged from −0.31 to 0.14, with all five studies reporting either decreased or unchanged BMD. The composite weighted mean effect size, *d*, was −0.26, and its CI was between −0.37 and −0.15. This implies that overall BMD was significantly lower in depressed than in nondepressed men (*P* < 0.001).

In four studies, only premenopausal women were examined. The effect size ranged from −0.28 to 0.18, with three studies reporting either decreased or unchanged BMD and one study showing increased BMD. However, the composite weighted mean effect size, *d*, was −0.14, and its CI was between −0.37 and −0.08. This implies that overall BMD was not significantly different in depressed and nondepressed women (*P* = 0.202). In two studies, only postmenopausal women were examined. There were no differences in BMD between the depressed and the nondepressed participants.

In four studies, depression was evaluated by an expert interview, and in one study, data on females and males were shown separately. When pooled for females and males, the effect sizes ranged from −0.31 to 0.18, with four studies reporting either decreased or unchanged BMD, and one study showing increased BMD. The composite weighted mean effect size, *d*, was −0.262, and its CI was between −0.39 and −0.13. This implies that overall BMD was significantly lower in depressed than in nondepressed participants (*P* < 0.001). For females, the effect sizes ranged from −0.282 to 0.18, with two studies reporting either decreased or unchanged BMD and one study showing increased BMD. However, the composite weighted mean effect size, *d*, was −0.14, and its CI was between −0.38 and 0.10. This implies that overall BMD was not significantly different in depressed women evaluated by an expert and nondepressed women (*P* = 0.252). For males, the effect size ranged from −0.31 to −0.29, with both studies reporting either decreased or unchanged BMD. The composite weighted mean effect size, *d*, was −0.31, and its CI was between −0.46 and −0.15. This implies that the overall BMD was significantly lower in depressed men evaluated by an expert than in nondepressed men (*P* < 0.001).

In seven studies, depression was evaluated by self‐reports. In one study, data on females and males were shown separately. When pooled for females and males, the effect sizes ranged from −0.22 to 0.00, with all studies reporting either decreased or unchanged BMD. The composite weighted mean effect size, *d*, was −0.10, and its CI was between −0.19 and −0.01. This implies that the overall BMD was significantly lower in depressed individuals evaluated by self‐reports than in nondepressed participants (*P* = 0.037). For females, the effect sizes ranged from −0.17 to 0.00, with all four studies reporting either decreased or unchanged BMD. However, the composite weighted mean effect size, *d*, was 0.00, and its CI was between −0.08 and 0.07. This implies that overall BMD was not significantly different in depressed women evaluated by self‐reports and nondepressed women (*P* = 0.937). For males, the effect sizes ranged from −0.21 to 0.14, with all three studies reporting either decreased or unchanged BMD. The composite weighted mean effect size, d, was −0.20, and its CI was between −0.36 to −0.04. This implies that overall BMD was significantly lower in depressed men evaluated by self‐reports than in nondepressed men (*P* = 0.012).

## Discussion

The meta‐analysis shows that major depressive disorder is associated with low bone density. Pooled data from men and women show that the bone density in all three examined locations (lumbar spine, femur, and total hip) was decreased with small‐to‐medium effect sizes. This result was similar to the conclusions made by the groups of Itai Bab and Brianna Mezuk (Mezuk et al. 2008; Yirmiya and Bab 2009). Of note, six new studies have been included since these reviews. No evidence for publication bias could be identified. The finding is of considerable importance, as osteoporosis and osteoporotic fractures may constitute a partial explanation for the increased mortality and decreased life expectancy in patients with severe mental disorders (Chang et al. 2011).

Examining men and women shows low BMD in the lumbar spine and femur in women and low BMD in the hip in men, which may correspond to a different pattern. However, the results may be an effect of the higher number of depressed women (1341) than depressed men (501) examined in the studies. The differences between men and women with MDD and the comparison group tended to be higher when examined by expert interviewers. Estimates of depression based on self‐ratings typically yield depression prevalence estimates that are considerably higher than the estimates based on expert interviews. This means that studies based on self‐rating may suffer from a high number of subjects false positively classified as depressed. There is no evidence that low BMD in depression is limited to any age group.

Although the phenomenon of decreased BMD in patients with depression is well established, the studies provide little evidence of the mediation or the genesis of osteoporosis. Only one of the included studies reported a significant correlation with cortisol (Altindag et al. 2007), one reported a significant effect of physical fitness (Diem et al. 2013b), two reported significant effects of smoking (Charles et al. 2012; Diem et al. 2013b), and none reported a significant effect of antidepressant medication on BMD. When evaluating cofactors, we must consider that most of the studies were pilot studies. In the 14 studies using expert interviews, the sample size of the depressed participants varied between 6 and 169. These sample sizes have the power to detect only medium‐to‐strong effect sizes and do not allow for multiple regression analysis with a high number of covariates.

Depression has an increased bidirectional association with a broad spectrum of medical disorders. Some of these disorders like thyroid disorders, diabetes, disorders with impaired vitamin D action, Cushing's syndrome, cancer and its treatment, chronic inflammatory disease, and its treatment with glucocorticoids or other substances, also directly affect bone. All studies included in this meta‐analysis report to have excluded men and women with comorbidity directly affecting bone. If we assume that this strategy was successful, the difference between healthy persons and men and women with depression in the general population may be underestimated. If we assume that it was incompletely effective, this may create a bias toward a higher difference. The latter possibility is not in accordance with the finding of similar BMD deficits in all age groups. All studies were controlled for antidepressant treatment. As discussed above the negative findings may be due to insufficient power.

The meta‐analysis was able to identify a substantial number of publications. We found no evidence for publication bias using the funnel plot technique. With respect to search bias, there was an excellent correspondence with earlier meta‐analyses. The main limitation is the pilot character of most studies included. A methodological limitation is the use of DEXA in all studies. Standard DEXA technique has only a limited sensitivity for changes in trabecular bone which are characteristic for the effects of glucocorticoids (Leib and Winzenrieth 2015).

## Conclusion

This meta‐analysis points to the following research needs. Longitudinal studies with a sufficient power to evaluate potential mediators of the effect of depression on BMD are needed. These studies should use expert interviews to reduce the high number of subjects with a false‐positive diagnosis of depression that occurs in studies using self‐reports and carefully assess the relevant covariates. Such studies may guide the future development of prophylactic or therapeutic strategies for low BMD in patients with depression.

## Conflict of Interest

None declared.
